# Yeast-derived low-purity FGF2 supports bovine ESC and MSC aggregates in suspension

**DOI:** 10.3389/fnut.2025.1679490

**Published:** 2025-11-28

**Authors:** Gaya Savyon, Shadi Tawil, Yuval Tadmor, Atar Shimshi, Yair Porat, Neta Agmon, Sharon Schlesinger, Tamir Tuller, Iftach Nachman

**Affiliations:** 1 School of Neurobiology, Biochemistry and Biophysics, Tel Aviv University, Tel Aviv, Israel; 2 Department of Animal Sciences, Faculty of Agriculture, The Hebrew University of Jerusalem, Rehovot, Israel; 3 BioDalia Microbiological Technologies Ltd., Dalia, Israel; 4 Department of Biomedical Engineering, Faculty of Engineering, Tel Aviv University, Tel Aviv, Israel; 5 Alagene Ltd., Rehovot, Israel

**Keywords:** FGF2, low-cost media, bovine mesenchymal stem cells, bovine embryonic stem cells, cell aggregate

## Abstract

Fibroblast Growth Factor 2 (FGF2) is an essential component of media for cultivated meat production, supporting the proliferation, survival, and differentiation of various cell types. The high cost of recombinant FGF2, typically produced in *Escherichia coli* (*E. coli*) with laborious downstream processing, remains a major bottleneck to commercial scalability. In this study, we developed a low-cost production platform using *Pichia pastoris (P. pastoris)*, a food-grade yeast capable of secreting properly folded proteins into the culture medium. We produced bovine FGF2 and purified it using tangential flow filtration (TFF), omitting chromatography to reduce processing complexity and cost. The biological activity of these low-purity FGF2 variants was assessed in two relevant models: bovine embryonic stem cells (bESCs) cultured as 3D aggregates under fully defined, animal component-free conditions, and immortalized bovine mesenchymal stem cells (bMSCs) in both 2D and 3D formats. Across all assays, the yeast-derived FGF2 variants matched or exceeded the performance of commercial high-purity FGF2 in promoting aggregate growth, mesodermal differentiation, and cell proliferation. Notably, both liquid and freeze-dried formulations of the TFF-purified FGF2 showed robust functionality, underscoring their suitability for industrial applications. These findings demonstrate that simplified, chromatography-free production of bioactive FGF2 is feasible and effective, offering a scalable and economically viable solution for next-generation cultivated meat media.

## Introduction

Fibroblast Growth Factor 2 (FGF2) is a critical signaling molecule widely used in the cultivated meat industry to support the expansion, survival, and lineage specification of various cell types. It plays essential roles in maintaining pluripotency and cell survival in embryonic stem cells (ESCs) (1, 2), enhancing proliferation and inhibiting senescence in mesenchymal stem cells (MSCs) (3–6), and supporting self-renewal of satellite cells (7). In addition, FGF2 is crucial during several stages of differentiation, including mesoderm commitment, paraxial mesoderm maturation, and myogenesis (8–10). These diverse functions underscore its widespread use across species and bioprocessing stages in the development of scalable cultivated meat production platforms (11, 12).

Cultivated meat production remains costly in part due to its reliance on recombinant FGF2, a key component of serum-free media. FGF2 alone can account for a substantial portion of media expenses, particularly in large-scale applications where sustained cell expansion is required (11, 12). Industrial production of FGF2 is often performed using *E. coli* expression systems. Numerous studies, including (13, 14) and Semper and Savchenko (13), have demonstrated that FGF2 can be expressed in *E. coli* in a soluble and bioactive form when appropriate chaperones, expression conditions, or fusion partners are applied. Nevertheless, large-scale bacterial systems rely on intracellular protein expression that necessitates cell-lysis–based recovery, which increases downstream complexity and cost relative to secretion-based hosts. At the industrial scale, these extra operations - cell disruption, clarification, and often refolding or tag-removal, represent significant time and cost bottlenecks.

Alternative recombinant protein production platforms developed in recent years may offer several advantages in this domain. One of them is based on *Pichia pastoris*, a methylotrophic yeast widely used for industrial-scale protein expression. *P. pastoris* offers several advantages over bacterial systems, including the ability to secrete properly folded proteins directly into the culture medium, thereby eliminating the need for cell lysis, refolding, or complex tag removal (15–19). This secretion-based expression system also enables simplified, non-chromatographic purification strategies such as TFF, which can dramatically reduce downstream processing costs. Leveraging these advantages, we explored whether yeast-expressed, minimally purified FGF2 could serve as a cost-effective alternative for use in cultivated meat media.

In this work, we develop processes for generation of recombinant FGF2 in *P. pastoris*. We evaluate the functional efficacy of the yeast-derived FGF2 variants in two biologically relevant cellular models: bESCs cultured as three-dimensional (3D) suspended aggregates and immortalized bMSCs. Across both models, we found that the low-purity FGF2 preparations produced in *P. pastoris* performed comparably to commercial high-purity FGF2 derived from *E. coli*. These results demonstrate that simplified, chromatography-free FGF2 production can effectively support key cellular processes relevant to cultivated meat, offering a scalable and economically viable alternative to conventional growth factor production.

## Results

### Bovine FGF2 production using yeast fermentation

The project goal was to minimize production costs for FGF2, making it more economically viable for the cultivated meat industry. This was addressed through two complementary strategies: selecting an optimal expression host and developing a cost-effective downstream fermentation and purification process.

As an expression host, we selected *P. pastoris* for the reasons explained above. To reduce costs in the downstream process, we implemented expression of recombinant proteins in *Pichia pastoris* TFF as the final purification method. TFF is a membrane-based purification and concentration technique in which the feed stream flows parallel to the membrane surface, reducing fouling and enabling continuous filtration. Unlike dead-end filtration, TFF allows fast flow filtration and is widely employed for industrial protein separation. Using ultrafiltration membranes with a molecular weight cut-off of 50–100 kDa allows separation of large MW proteins, thereby facilitating the partial purification of FGF. Because *P. pastoris* secretes FGF2 into the culture medium, TFF provides an efficient chromatography-free strategy suitable for low-cost, scalable downstream processing (20). Prior to TFF, the culture medium underwent two initial clarification steps: centrifugation to remove cells, followed by pre-filtration through 0.22 μm membranes. These steps are essential not only for TFF but also for column-based purification workflows. The compatibility of *P. pastoris* with secretion-based expression enabled us to apply TFF directly to the culture medium, streamlining the workflow by eliminating the need for cell lysis and complex purification steps.

**FIGURE 1:**
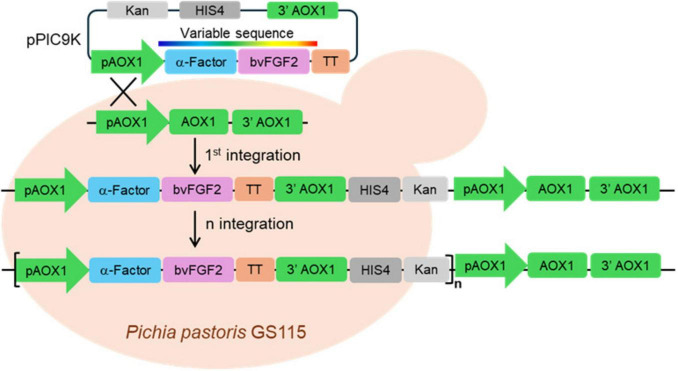
Schematic representation of the *P. pastoris* bvFGF2 expression cassette and integration strategy.

The bovine FGF2 (bvFGF2) expression cassette was cloned into pPIC9K vector downstream of the *AOX1* promoter sequence (*pAOX1*). Following linearization, the construct was integrated into native AOX1 locus in the *P. pastoris* genome by homologous recombination. In some of the generated strains, multiple integration events occurred, potentially enhancing expression levels. For further genetic optimization, the expression cassette was engineered to include an α-factor secretion signal, the bvFGF2 coding sequence, and the transcriptional terminator (TT) (Figure 1). The FGF2 coding sequence itself was optimized using computational modeling approaches to enhance translational efficiency and expression stability in *P. pastoris*, as described in Materials and methods section.

To evaluate the feasibility of this production pipeline, we generated and compared three versions of FGF2 using this system: (1) FGF2 purified using a 100 kDa filter, (2) FGF2 purified using a 50 kDa filter, and (3) FGF2 that purified using a heparin affinity column. When only filtration was used, the purity of the FGF2 protein reached up to 5% of the total protein content in the eluted fraction. In contrast, heparin column purification yielded a highly pure product, with protein purity exceeding 97%. All FGF2 samples had low endotoxin levels, with concentrations not exceeding 0.5 EU/ml. We also compared the biological activity of FGF2 in two physical forms: a concentrated liquid solution and a freeze-dried powder. Freeze dry does not affect purity level but affects end user experimental practice. To assess the broader utility of these FGF2 preparations in cultivated meat production, we tested them in two biologically relevant models for the cultivated meat industry: bESC-derived aggregates and bMSC 2D and 3D culture.

### Low-purity bFGF2 supports bESC-based aggregate growth and mesodermal differentiation

**FIGURE 2:**
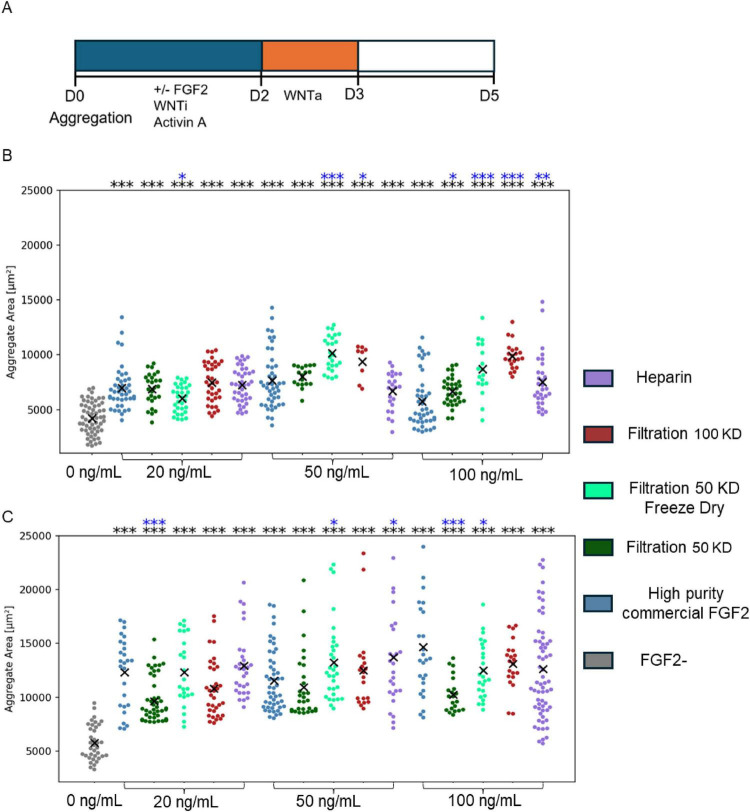
Yeast-derived low-purity FGF2 support bESC-based aggregates growth. **(A)** Schematic representation of the experimental timeline. **(B,C)** Aggregate area on day 3 **(B)** and day 5 **(C)** under different FGF2 conditions. Each dot represents a single aggregate. X marks indicate the mean aggregate area per condition. Data are pooled from two technical replicates (two wells per condition, each containing 20 > aggregates). FGF2 was applied at three concentrations: 20, 50, and 100 ng/mL, as indicated below the x-axis. Statistical comparisons were performed using unpaired two-tailed *t*-tests. Significance levels: *P < 0.05, **P < 0.01, ***P < 0.001, when compared to the commercial FGF2 (blue * marks) or to the FGF2- (black * marks) condition.

We first tested the ability of the FGF2 variants to support bESC-based aggregate formation and growth in suspension under serum-free conditions. Notably, bovine ESCs are notoriously difficult to culture, requiring tightly controlled conditions and costly, fully defined media formulations. Aggregates were formed by seeding single-cell suspensions into uncoated wells and culturing under continuous orbital shaking. During the first 2 days of culture, the aggregates were exposed to one of the following FGF2 conditions: *P. pastoris* derived FGF2 filtered at 100 KDa, 50 KDa, heparin column, or 50 KDa freeze-dried, positive control (commercial native, non-mutated and non-heat-stable high-purity FGF2 derived from *E. coli*) and negative control (no FGF2). Each FGF2 variant was tested at three concentrations: 20 ng/mL, 50 ng/mL, and 100 ng/mL, and for each condition, two biological replicates were analyzed. After 2 days of aggregation, the medium was replaced with a differentiation medium (not containing FGF2) in which the aggregates were cultured for an additional 3 days (Figure 2A).

Aggregate size was quantified on Day 3 and 5. All FGF2-treated conditions supported significant aggregate growth compared to the no-FGF2 control (Figures 2B,C). Notably, the yeast-derived FGF2 variants were comparable to, and in some cases outperformed, the commercial control in terms of aggregate size progression.

As the TFF-purified FGF2 performed similarly to the heparin-purified variant and its production is more cost-efficient, we conducted the differentiation analysis using the TFF-purified variants together with the commercial FGF2 and FGF2-free controls. Additionally, as the freeze-drying process did not appear to impact FGF2 functionality, we limited the differentiation analysis to the liquid formulations of each variant.

**FIGURE 3:**
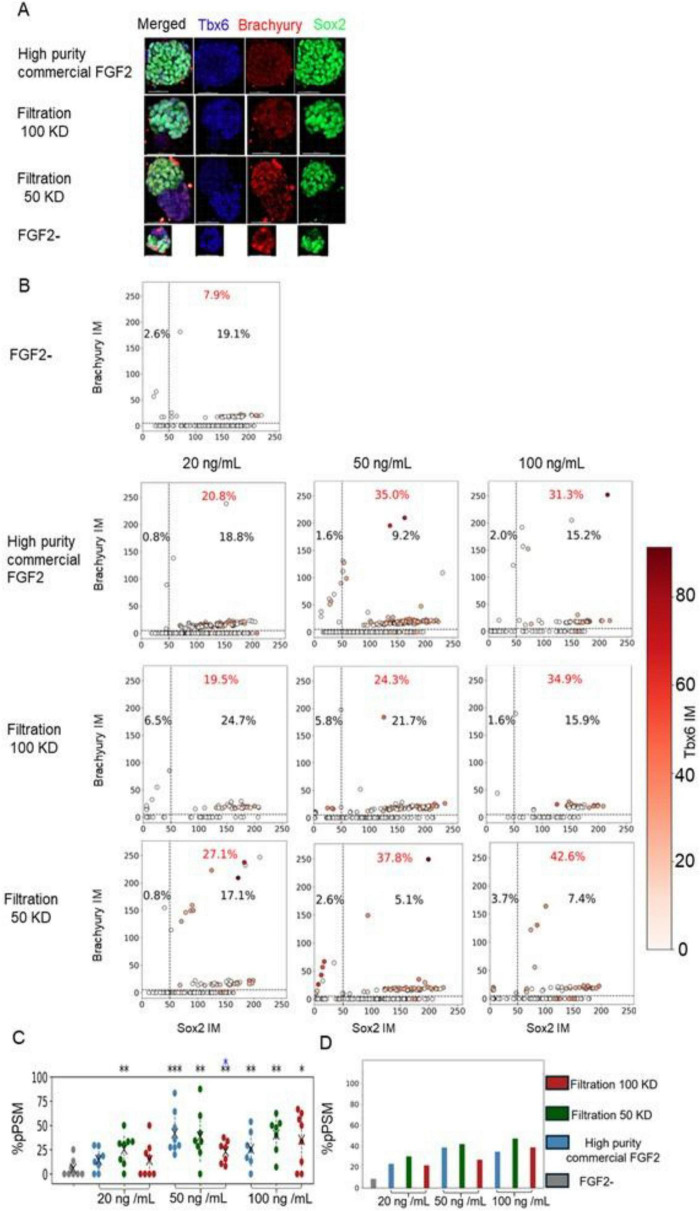
Yeast-derived low-purity FGF2 supports mesodermal differentiation of bESC-based aggregates. **(A)** Immunofluorescence images of aggregates at day 5 stained for Sox2 (green), Tbx6 (blue) and Brachyury (red), under different FGF2 variants at 50 ng/mL. Scale bar = 50 μm. **(B)** Single cell levels of Sox2, Brachyury and Tbx6 in day 5 aggregates cultured in suspension under different FGF2 conditions. Each dot represents a single cell, with Tbx6 expression level indicated by shades of red. Dashed lines mark the thresholds used to define specific cell populations. For each condition, two technical replicates (two wells) were performed, each containing at least 20 aggregates. From these, eight aggregates per condition were randomly selected for single-cell analysis. Each aggregate contained dozens of cells. Data are pooled from all eight aggregates per condition without separation between aggregates. Proportions (%) of cells falling within three defined groups are indicated: Cells classified as pPSM (expressing Tbx6 above the threshold, red); Cells classified as early mesoderm, defined as Brachyury-high, Sox2-low, and Tbx6-low (top left quadrant). Cells classified as NMPs, expressing both Sox2 and Brachyury above the thresholds (top right quadrant). **(C,D)** Quantification of the proportions of NMPs, early mesoderm, and pPSM cells at day 5 across different FGF2 conditions. **(C)** Percentage of pPSM cells per aggregate. Each dot represents a single aggregate (each dot = one aggregate; *n* = 8 aggregates per condition), color-coded by FGF2 condition. Statistical comparisons were performed using the Mann–Whitney U test. *P < 0.05, **P < 0.01, ***P < 0.001, when compared to the commercial FGF2 (blue * marks) or to the FGF2- (black * marks) condition. **(D)** Percentage of pPSM cells from pooled single-cell data across all aggregates in each condition.

To evaluate whether the yeast-derived low-purity FGF2 variants (Filtration 50 KD and Filtration 100 KD) support mesodermal differentiation of bESC aggregates, we analyzed the expression of three lineage markers at the single-cell level: Sox2, Brachyury, and Tbx6. Sox2, when not co-expressed with Brachyury, is associated with pluripotency and neuroectodermal identity, while Brachyury marks early mesodermal progenitors. Cells co-expressing Sox2 and Brachyury are classified as neuromesodermal progenitors (NMPs), a bipotent population found in the posterior embryo that can give rise to the neural tube and advanced mesodermal populations such as intermediate mesoderm and the paraxial mesoderm. Tbx6, in a specific marker for posterior paraxial mesoderm (pPSM), a more advanced cell fate along the differentiation trajectory from embryonic stem cells toward the skeletal muscle lineage. This developmental progression is highly conserved among vertebrates and has been extensively reviewed (21, 22). Representative immunofluorescence images illustrating the expression patterns of Sox2, Brachyury, and Tbx6 on Day 5 are shown in Figure 3A. These images provide qualitative confirmation that the tested FGF2 variants support the emergence of key developmental lineages. To quantitatively assess lineage specification, we performed single-cell fluorescence analysis across individual aggregates. This approach allowed us to identify distinct cell populations corresponding to NMPs, early mesoderm, and pPSM, as defined by specific combinations of Sox2, Brachyury, and Tbx6 expression (Figure 3B). To quantitatively compare mesodermal differentiation outcomes across conditions, we analyzed the proportions of NMPs, early mesoderm, and pPSM populations in each aggregate on day 5 (Figure 3B).

We find that both yeast-derived low-purity FGF2 variants (50 and 100 kDa purifications) effectively supported mesodermal specification of bESCs (Figures 3C,D). In most cases, their performance was statistically indistinguishable from that of the high-purity commercial FGF2, and in some conditions, they even exceeded its effectiveness.

In summary, our findings demonstrate the potential of the low-cost, yeast-derived FGF2 to effectively replace traditional high-purity FGF2 in supporting early growth and differentiation of bESC aggregates under defined, suspended culture conditions.

### Low purity Pichia-derived bFGF2 enhances bMSC growth in 2D and 3D systems

We next evaluated the biological functionality of the novel yeast-derived low-purity FGF2 variants in an additional model system: immortalized bMSCs. bMSCs are a highly relevant cell type for the cultivated meat industry due to their robust proliferative capacity, and ability to differentiate into muscle and fat lineages.

**FIGURE 4:**
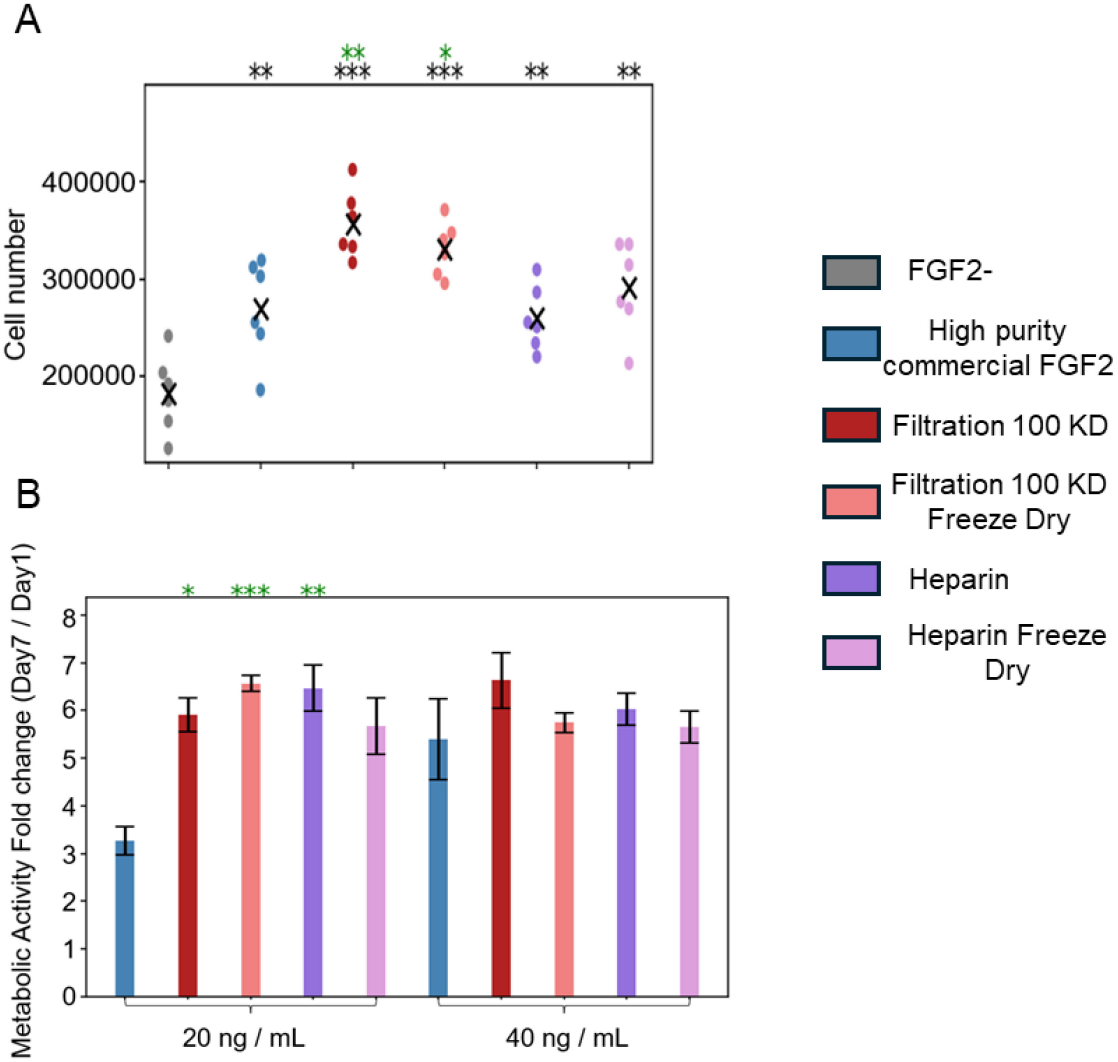
Yeast-derived low-purity FGF2 enhances bMSC proliferation and metabolic activity in 2D and 3D. **(A)** Cell number after 4 days in culture with different FGF2 conditions. Each dot represents a well and color-coded by FGF2 condition. X marks indicate the mean value. Six technical replicates (wells) were performed per condition. Statistical comparisons were performed using the Mann–Whitney U test; *P < 0.05, **P < 0.01, ***P < 0.001, compared to the commercial FGF2 (green * marks) or to the FGF2-free (black * marks) condition. **(B)** Fold-change in metabolic activity of bMSCs from days 1 to 7, grown with different FGF2 conditions, as measured by resazurin. Changes in fluorescence were normalized to media without cells and values are shown as mean ± SD. Two technical replicates per condition were performed within each of three independent experiments. Statistical comparisons were performed using the Mann–Whitney U test, significance codes as above.

To assess the mitogenic potential of the yeast-derived FGF2 variants under nutrient-limited conditions, we cultured immortalized bMSCs for 4 days in medium supplemented with 3% FBS, commercial heat-stable high purity *E. coli* derived FGF2, or one of four novel Pichia-derived FGF2 preparations (Figure 4A). All FGF2-supplemented conditions significantly enhanced cell numbers compared to the corresponding no FGF2 control (*p* < 0.0001). Specifically, both liquid and freeze-dried versions of the 100 kDa filtration-purified FGF2 led to cell counts equivalent to or exceeding those obtained with commercial FGF2 (*p* < 0.01 for 100KD and *p* < 0.05 for the freeze-dried version). The heparin-purified variants promoted proliferation, similar to the commercial heat-stable FGF2. These results demonstrate that low-purity Pichia-derived FGF2 formulations are effective in supporting bovine MSC expansion in 2D even under serum-restricted conditions.

We next tested the functional impact of FGF2 variants in a bMSC-based 3D culture context, relevant for tissue engineering and cultivated meat applications. bMSCs were seeded at 100,000 cells/mL and cultured in suspension on an orbital shaker for 7 days with 20 ng/mL or 40 ng/mL of FGF2 (Figure 4B). To avoid inaccuracies in cell quantification due to incomplete or excessive dissociation, we employed the redox-sensitive dye resazurin to monitor metabolic activity as an indirect measure of cell proliferation. In all FGF2-treated conditions, a progressive increase in resazurin signal was observed from day 1 to 7, indicating enhanced metabolic activity consistent with cell expansion. In contrast, cultures lacking FGF2 did not exhibit a measurable increase in signal and are therefore not shown. All novel FGF2 treatments led to a significant increase in metabolic activity compared to commercial FGF2 at the 20 ng/mL dose, with the 100 kDa freeze-dried formulation showing the greatest fold change (*p* < 0.0001). Notably, all novel FGF2 variants at 20 ng/mL achieved metabolic activity levels comparable to those observed with 40 ng/mL of commercial FGF2, suggesting that the bioactivity of these formulations may be approximately twice as potent. At the 40 ng/mL dose novel formulations induced similar growth levels to the commercial control. This may indicate that a saturation point for FGF2-stimulated proliferation is reached under these 3D culture conditions. These findings confirm that low-purity *P. pastoris*-derived FGF2 is functionally active and supports robust 3D bMSC culture growth, with potential applications in scalable bioprocesses.

## Discussion

This study demonstrates that low-purity FGF2 produced in Pichia pastoris using a simplified, chromatography-free process can effectively support key cellular processes required for cultivated meat production.

A central design consideration of this study was the selection of an expression host and purification strategy that together enable efficient, low-cost production of bioactive FGF2 suitable for food applications. We selected *P. pastoris* as the expression system for several key reasons. Many biotechnological products expressed in *P. pastoris* have received GRAS (Generally Recognized As Safe) status from regulatory authorities (23), supporting its suitability for food-related use. Its genome is fully sequenced, facilitating precise genetic manipulation (24). It enables high-yield recombinant protein production even on minimal substrates and can secrete active proteins directly into the medium (25). This secretion capability eliminates the need for cell lysis and the extensive clarification and refolding operations required for intracellular expression systems. Collectively, these characteristics make *P. pastoris* a more efficient and cost-effective alternative to commonly used expression hosts for FGF2 production.

In addition to the advantages of the yeast host, the TFF approach used in this study provided substantial process-level benefits. TFF offers significant economic advantages over traditional chromatography or HPLC, reducing purification costs by at least 50%. The cassettes are less expensive, the filtration process is faster, and the simplified workflow lowers labor requirements. When combined with the secretion-based expression of *P. pastoris*, this method minimizes downstream complexity and overall production costs while maintaining strong protein functionality.

Across two biologically relevant models, bESC aggregates and immortalized bMSCs, the yeast-derived FGF2 variants matched or exceeded the performance of commercial high-purity FGF2 in supporting aggregate formation, cell proliferation, and mesodermal differentiation. These findings establish that high-cost purification is not a prerequisite for biological efficacy, and position low-purity, yeast-derived FGF2 as a promising alternative for cost-sensitive bioprocesses in the cultivated meat industry.

A key innovation of this work lies in the use of *P. pastoris* as an expression platform combined with TFF for downstream processing (Figure 1). This system eliminates the need for cell lysis, protein refolding, and chromatography, which are required in conventional systems that relies on intracellular expression for the production pipelines and contribute substantially to the cost. To substantiate this cost advantage, we performed an internal techno-economic comparison of our production stages. While the upstream fermentation costs were similar between yeast- and bacteria-based systems, the downstream stage showed a major divergence. When using TFF filtration, downstream processing costs were approximately twice the upstream cost, whereas heparin-based chromatographic purification increased downstream costs to roughly six times the upstream cost. Overall, the filtration-based workflow therefore reduced total production costs by about 64% relative to chromatography. Notably, even without chromatographic purification, the resulting FGF2 preparations, with approximately 5% purity of total proteins, retained strong biological activity across all tested assays. Additionally, the compatibility of these variants with freeze-drying provides a practical advantage for storage and transport, further enhancing their industrial scalability. Taken together, these process simplifications offer a compelling strategy for reducing growth factor costs, a major barrier to economically viable cultivated meat production.

The biological performance of yeast-derived low-purity FGF2 was evaluated across distinct stem cell types and culture formats. In the bESC model, all TFF-purified variants supported robust aggregate formation and growth under fully defined, animal component-free conditions (Figures 2B,C), and promoted mesodermal lineage specification as shown by single-cell marker analysis (Figure 3). Similarly, in bMSCs, the 100 kDa and freeze-dried variants strongly enhanced cell proliferation and metabolic activity in both 2D and 3D contexts (Figure 4), often outperforming the commercial high-purity FGF2. Interestingly, we did not observe a clear dose-dependent response in bESC aggregate size progression for any of the tested FGF2 variants, including the commercial control. However, when evaluating other biological readouts, including the number of bESC aggregates formed per well after 3 days of culture (Supplementary Figure 1), and the metabolic activity of bMSC-based aggregates (Supplementary Figure 2) a dose-dependent effect was evident for the commercial FGF2. Notably, the low-purity, yeast-derived FGF2 variants produced comparable or even superior results in these assays compared to high concentrations of commercial FGF2.

These findings have important implications for the design of affordable and scalable media formulations in the cultivated meat industry. The demonstrated efficacy of low-purity FGF2, both in concentrated liquid solution and freeze-dried formats, suggests that media containing crude or partially purified growth factors can still achieve high biological performance when properly optimized. This opens the door to reformulating serum-free media using industrial-grade inputs that are fit-for-purpose rather than research-grade. In particular, the ability to support both pluripotent and mesenchymal stem cell cultures using cost-effective FGF2 aligns with the sector’s urgent need to reduce production costs without compromising consistency or safety.

While the results presented here highlight the strong potential of yeast-derived low-purity FGF2, several limitations and avenues for further development should be acknowledged. First, although the TFF-purified formulations were highly effective, their exact composition remains not fully characterized, and the presence of co-purified yeast proteins may introduce batch-to-batch variability. However, the use of *P. pastoris* as the expression host mitigates potential safety concerns, as it is widely accepted for food-related applications and many *P. pastoris* -based processes have received GRAS status. As a follow-up to this point, it is plausible that low-purity FGF2 preparations produced in other expression systems, such as *E. coli* or CHO cells, could exhibit distinct biological effects due to differences in their co-purified protein profiles and residual media components. Exploring these systems in future studies could provide valuable insight into how host-dependent factors influence the composition and bioactivity of crude FGF2 preparations. Nevertheless, detailed proteomic and functional analyses of these preparations will be important to ensure consistency and compliance with future regulatory frameworks. Additionally, while bESCs and immortalized bMSCs provide highly relevant models for the cultivated meat industry models, further validation in more cell types and organisms will be essential to confirm the generalizability of these findings.

In summary, this study presents a scalable, low-cost, and food-compatible platform for producing bioactive FGF2 using *P. pastoris* and TFF. Our results demonstrate that low-cost, chromatography-free FGF2 variants maintain comparable biological performance to high-purity FGF2, supporting cell proliferation, aggregate growth, and mesodermal differentiation in cultivated meat relevance models. These findings underscore the feasibility of using simplified, industrial-grade inputs to reduce the cost of growth factor supplementation, a major economic bottleneck in the field. The yeast-derived FGF2 platform introduced here represents a meaningful step toward more affordable, scalable, and commercially viable biomanufacturing solutions for next-generation food systems.

## Materials and methods

### Gene expression optimization based on computational tools

The coding sequence of FGF2 and the sequences surrounding it were optimized by various computational models to obtain high protein expression in Pichia. Among others, we introduced various patterns and motifs to improve translation initiation (26–28); in addition, we optimized the codon of FGF2 to fit them to *P. pastoris* and to decrease mutations rate and thus increase genetic stability (29). More specifically, we use the Evolutionary stability optimizer (ESO) algorithm (29) to remove short and long nucleotide repeats while choosing codons with high genomic frequency. Short nucleotide repeats may cause the slippage of the DNA polymerase and thus promote mutations. Longer nucleotide repeats may cause DNA recombination and thus also promote longer deletions/insertion and genomic instability. Codons with higher genomic frequency usually tend to be recognized by tRNAs with higher concentration, promoting higher translation efficiency; such codons may also optimize other aspects of gene expression (30). To optimize translation initiation efficiency, we introduced a context sequence surrounding the start codon that appears endogenous start codon of *P. pastoris* (28). This sequence is expected to promote the recognition of the start codon by the small sub-unit of the ribosome. In addition, we chose the first 10 codons such that the local mRNA folding energy in this region will be as high as possible (i.e., the mRNA will be less folded); this is expected to increase the accessibility of the small subunit of the ribosome to the start codon and improve translation initiation (26, 31).

### FGF2 production, fermentation, and purification

#### Fermentation process

FGF2 production in *P. pastoris* was performed as an aerobic batch fermentation consisting of two main stages: biomass accumulation and methanol-induced protein expression. Cells were first grown for 24 h under selective conditions in glycerol-based medium to promote biomass formation. Protein production was then induced for an additional 48 h by the addition of methanol as the sole carbon source, activating the AOX1 promoter that drives FGF2 expression. The fermentation was conducted under aerobic conditions with controlled pH and dissolved oxygen levels to support high cell density.

#### Purification process

Following completion of the induction phase, the culture was harvested and clarified in two sequential steps. First, biomass was removed by continuous centrifugation to separate yeast cells. The supernatant was then passed through a 0.22 μm membrane filter to further remove residual particulates and colloids. The resulting clarified medium contained the secreted FGF2 together with other extracellular yeast proteins and soluble medium components.

FGF2 purification was carried out using a high-flow TFF process. The clarified medium was filtered through membranes with molecular weight cut-offs of either 50 kDa or 100 kDa. The absence of a cell lysis step significantly reduced the load of insoluble debris and soluble contaminants that typically clog filtration membranes, allowing stable and continuous TFF operation. In selected experiments, a heparin-affinity purification step was performed as a benchmark for high-purity material. This method takes advantage of FGF2’s natural heparin-binding domain, which mediates strong and specific electrostatic interactions with heparin sulfate groups, enabling > 97% purity in a single step without engineered affinity tags.

#### Purity assessment

The purity of the yeast-derived FGF2 preparations was evaluated using SDS-PAGE followed by image-based quantification. Protein gels were imaged using a Bio-Rad ChemiDoc™ Imaging System, and the intensity of the FGF2 band was quantified relative to total lane intensity to estimate purity. The low-purity TFF-derived preparations contained approximately 5% FGF2 of total protein, whereas the heparin-purified preparations exceeded 97% purity.

#### Liquid formulation composition

The liquid solution obtained after TFF contained the secreted FGF2 together with residual medium components and co-secreted yeast proteins. No elution buffers, denaturing agents, or additives were used, as the TFF process relies purely on size-based separation rather than chemical affinity. In contrast, the heparin-purified FGF2 solution contained traces of the standard salt-based elution buffer used for column chromatography. For the freeze-dried samples, trehalose was added as a stabilizer to protect the protein during lyophilization and to preserve bioactivity upon reconstitution.

### bESC cell culture and maintenance

bESCs were cultured under serum-free conditions. The A-bESC line used in this study was originally established by the Alberio laboratory, as described by Kinoshita et al. (32). To maintain pluripotency, bESC were cultured in 2D on mitotically inactivated mouse embryonic fibroblasts (iMEFs) seeded on tissue culture-treated plastic plates coated with 0.2% gelatin. Cultured were maintained at maintained at 37 °C with 5% CO2. The basal medium was N2B27 supplemented with 20 ng/mL Activin A (338-AC, R&D Systems), 2 μM XAV939 (a Wnt pathway inhibitor, X3004, Merck), and 12.5 ng/mL heat-stable FGF2 (HS-FGF2, 100-18BHS, ThermoFisher). Cultures were passaged upon reaching approximately 80% confluence using TrypLE Express, and medium was replaced daily to support optimal cell growth and maintenance of pluripotency.

### bESC based aggregate formation and differentiation

To initiate aggregate formation, bESCs were dissociated into single cells using TrypLE Express or Accutase, as done for routine passaging. Cells were seeded into uncoated, feeder-free tissue culture plates and maintained in suspension on an orbital shaker at 125 rpm. Aggregation was carried out in the pluripotency maintenance medium (N2B27 supplemented with Activin A and a Wnt inhibitor), with FGF2 added or omitted according to the tested condition. In these experiments, the commercial FGF2 used as a positive control differed from the one used for 2D pluripotency maintenance, it was replaced with native FGF2 (100-18B, Thermo Fisher) instead of the heat-stable variant. The seeding density was 65,000 cells/mL.

After 2 days of aggregation (day 0–2), differentiation was induced by replacing the medium with N2B27 supplemented with 10 μM CHIR99021 (a Wnt agonist) for 24 h. On day 3, the CHIR-containing medium was replaced with plain N2B27 without additional supplements and maintained until day 5. Aggregates remained in suspension under continuous shaking throughout the entire 5-day culture period.

### Aggregate size analysis

Live aggregates were imaged on days 3 and 5 using an epifluorescence microscope in brightfield mode. To ensure complete sampling, the entire well was imaged using a tiled acquisition approach with automated stitching, allowing all aggregates within each well to be captured and analyzed. Aggregate size was quantified using FIJI (ImageJ) by manually outlining the perimeter of each aggregate and measuring the projected 2D area in square microns (μm^2^).

For statistical analysis of aggregate area distributions, *t* tests were performed.

Aggregate size was used as a proxy for growth, based on common practice in 3D stem cell culture systems, where changes in size often correlate with changes in total cell number. In our system, single-cell immunostaining and imaging of multiple aggregates per condition did not reveal prominent lumen formation or internal cavities, supporting the interpretation that increased size reflects higher cell number.

### Immunofluorescence analysis

Aggregates from each experimental condition were pooled and fixed in 4% paraformaldehyde, either overnight at 4 °C or for 1 h at room temperature. Fixed aggregates were then blocked and permeabilized overnight at 4 °C in PBS containing 7.5% fetal bovine serum and 0.2% Triton X-100.

Primary antibody incubation was performed for 2–3 days at 4 °C in blocking buffer. The following primary antibodies were used: anti-Brachyury (AF2085, R&D Systems) at 1:10 dilution, anti-Sox2 (149881182, Thermo Fisher) at 1:200 dilution, and anti-Tbx6 (ab38883, Abcam) as 1:200 dilution. After incubation, aggregates were washed twice in blocking buffer and incubated again in blocking solution overnight. Secondary antibodies were then applied overnight at 4 °C at a dilution of 1:500.

Following staining, aggregates were washed and transferred to glass-bottom plates, embedded in 0.5% low-melting-point agarose, and imaged using a confocal microscope. Z-stacks were acquired in two fluorescence channels.

Image analysis was performed using Imaris. Stained cells were segmented in 3D to extract their Euclidean (x, y, z) coordinates and mean fluorescence intensity in each channel. This data was used to assign marker expression levels across aggregates.

### 3 D culture of immortalized bovine mesenchymal stem cells

Immortalized bMSCs, generated via hTERT transduction, were cultured in standard two-dimensional conditions using DMEM supplemented with 10% fetal bovine serum (FBS), 1% penicillin-streptomycin, and 1% L-glutamine. Prior to initiating 3D culture, cells were dissociated into single-cell suspensions using TrypLE Express. For 3D aggregation, 100,000 cells were seeded per well in 12-well plates that had been pre-treated with a commercially available anti-adherent solution, ensuring prevention of cell attachment and promoting spheroid formation.

Cells were cultured for 7 days in DMEM supplemented with 5% FBS, either alone or in combination with FGF2. The following FGF2 variants were tested: commercial heat-stable bovine FGF2 and four *P. pastoris*-derived formulations: (1) filtration-purified 100 kDa (liquid), (2) freeze-dried filtration-purified 100 kDa, (3) heparin-purified (liquid), and (4) freeze-dried heparin-purified. All FGF2 variants were tested at concentrations of 10 ng/mL, 20 ng/mL, or 40 ng/mL. No media changes were performed during the 7-day culture period.

To evaluate cell viability and proliferation, metabolic activity was assessed on day 1 and 7 using the Alamar Blue assay. Fluorescence was measured at 560/590 nm using a multimode plate reader. All values were blank-subtracted, and fold change in metabolic activity was calculated by normalizing day 7 measurements to baseline day 1 values. Each condition was tested in two to three technical replicates across independent experiments.

### 2 D Proliferation assay

To evaluate the proliferative effect of the *P. pastoris*-derived FGF2 variants under serum-reduced conditions, immortalized bMSCs were seeded at a density of 50,000 cells per well in standard tissue culture-treated 12-well plates. Cells were cultured for 4 days in DMEM supplemented with either 3% or 5% FBS, in the presence or absence of 10 ng/mL of FGF2. The FGF2 conditions included a commercial heat-stable bovine FGF2 and four novel variants: filtration-purified 100 kDa (liquid), freeze-dried filtration-purified 100 kDa, heparin-purified (liquid), and freeze-dried heparin-purified.

At the experimental endpoint, cells were dissociated using TrypLE Express and total live cell numbers were quantified using the Countess II Automated Cell Counter (Thermo Fisher Scientific) following the manufacturer’s instructions. Each condition was performed in biological triplicates. Mean cell counts were calculated for each treatment, and proliferative effects were compared to serum-only controls and the commercial FGF2-treated group using one-way ANOVA followed by multiple comparisons tests.

### Alamar blue metabolic activity assay

Cell metabolic activity was assessed using the Alamar Blue reagent to evaluate viability and relative growth in 3D culture. On days 1 and 7 of the 3D aggregation assay, Alamar Blue was added directly to each well at 10% of the total culture volume and incubated for 4 h at 37 °C. Fluorescence was measured at an excitation/emission wavelength of 560/590 nm using a multimode microplate reader.

All fluorescence values were corrected for background by subtracting blanks (medium with reagent, no cells). For each well, the day 7 measurement was normalized to its corresponding day 1 value to calculate fold change in metabolic activity. Results are presented as mean ± standard deviation across technical replicates. Increased fold change was interpreted as an indicator of enhanced cell viability and growth within the 3D aggregates.

### bMSC statistical analysis

For each FGF2 condition, six technical replicates (six wells) were analyzed in the 2D proliferation assay, and two technical replicates per condition were performed within each of three independent experiments for the 3D Alamar Blue assay.

Statistical significance was evaluated using the Mann–Whitney U test for pairwise comparisons between treatment and control groups. Significance levels are indicated in figures as follows: *p < 0.05, **p < 0.01, ***p < 0.001, ****p < 0.0001, with green asterisks denoting comparisons to the commercial FGF2 and black asterisks denoting comparisons to the FGF2-free control.

## Data Availability

The raw data supporting the conclusions of this article will be made available by the authors, without undue reservation.

## Supplementary material

Supplementary Figure 1 FGF2 version and concentration effect bESC-based aggregates growth in shaking conditions. **(A)** Schematic representation of the experimental timeline. **(B)** Quantification of the number of aggregates per well on day 3, comparing different FGF2 concentrations (ng/mL) and versions. Aggregates were cultured in shaking conditions. **(C)** Quantification of aggregate area (μm^2^) on day 3 of differentiation under different FGF2 concentrations and versions. Aggregates were cultured in suspension with either heat-stable FGF2 (HS-FGF2) or standard FGF2, both commercially available at high purity. Error bars indicate standard deviation. Quantifications in both panels were performed based on bright-field images acquired from each well and analyzed using FIJI.

Supplementary Figure 2 Fold change in metabolic activity at day 7 in 3D-cultured bMSC with increasing commercial FGF concentrations. Cells were seeded in 3D at densities of 50K, 100K, or 200K and treated with 0 (control), 10, 20, or 40 ng/mL of growth factor. Alamar Blue fluorescence was measured on day 7 and normalized to values from day 2 for each seeding density. Bars represent mean ± standard deviation (SD) from 2 to 3 replicates per condition. Significant increases in normalized Alamar Blue signal were observed at 100K with 20 ng/mL (****P* = 0.0003) and 40 ng/mL (*****P*<0.0001), and at 200K with 10 ng/mL (**P* = 0.0475), compared to their respective controls (Sidak’s multiple comparisons test, α = 0.05).

## References

[1] Eiselleova L Matulka K Kriz V Kunova M Schmidtova Z Neradil J A complex role for FGF-2 in self-renewal, survival, and adhesion of human embryonic stem cells. *Stem Cells.* (2009) 27:1847–57. 10.1002/stem.128 19544431 PMC2798073

[2] Mossahebi-Mohammadi M Quan M Zhang J-S Li X. FGF signaling pathway: a key regulator of stem cell pluripotency. *Front Cell Dev Biol.* (2020) 8:79. 10.3389/fcell.2020.00079 32133359 PMC7040165

[3] Ahn H-J Lee W-J Kwack K Kwon YD. FGF2 stimulates the proliferation of human mesenchymal stem cells through the transient activation of JNK signaling. *FEBS Lett.* (2009) 583:2922–6. 10.1016/j.febslet.2009.07.056 19664626

[4] Bianchi G Banfi A Mastrogiacomo M Notaro R Luzzatto L Cancedda R Ex vivo enrichment of mesenchymal cell progenitors by fibroblast growth factor 2. *Exp Cell Res.* (2003) 287:98–105. 10.1016/S0014-4827(03)00138-1 12799186

[5] Ito T Sawada R Fujiwara Y Seyama Y Tsuchiya T. FGF-2 suppresses cellular senescence of human mesenchymal stem cells by down-regulation of TGF-beta2. *Biochem Biophys Res Commun.* (2007) 359:108–14. 10.1016/j.bbrc.2007.05.067 17532297

[6] Tsutsumi S Shimazu A Miyazaki K Pan H Koike C Yoshida E Retention of multilineage differentiation potential of mesenchymal cells during proliferation in response to FGF. *Biochem Biophys Res Commun.* (2001) 288:413–9. 10.1006/bbrc.2001.5777 11606058

[7] Johnson SE Allen RE. Activation of skeletal muscle satellite cells and the role of fibroblast growth factor receptors. *Exp Cell Res.* (1995) 219:449–53. 10.1006/excr.1995.1251 7641796

[8] Chal J Oginuma M Al Tanoury Z Gobert B Sumara O Hick A Differentiation of pluripotent stem cells to muscle fiber to model Duchenne muscular dystrophy. *Nat Biotechnol.* (2015) 33:962–9. 10.1038/nbt.3297 26237517

[9] Ciruna B Rossant J. FGF signaling regulates mesoderm cell fate specification and morphogenetic movement at the primitive streak. *Dev Cell.* (2001) 1:37–49. 10.1016/s1534-5807(01)00017-x 11703922

[10] Yamaguchi TP Harpal K Henkemeyer M Rossant J. fgfr-1 is required for embryonic growth and mesodermal patterning during mouse gastrulation. *Genes Dev.* (1994) 8:3032–44. 10.1101/gad.8.24.3032 8001822

[11] Specht L. *An Analysis of Culture Medium Costs and Production Volumes for Cultivated Meat.* Washington, DC: The Good Food Institute (2020).

[12] Swartz E. *Anticipated Growth Factor and Recombinant Protein Costs and Volumes Necessary for Cost-Competitive Cultivated Meat.* Washington, DC: The Good Food Institute (2023).

[13] Semper C Savchenko A. Protein expression and purification of bioactive growth factors for use in cell culture and cellular agriculture. *STAR Protocols.* (2023) 4:102351. 10.1016/j.xpro.2023.102351 37314918 PMC10277608

[14] Venkatesan M Semper C Skrivergaard S Di Leo R Mesa N Rasmussen MK Recombinant production of growth factors for application in cell culture. *IScience.* (2022) 25:105054. 10.1016/j.isci.2022.105054 36157583 PMC9489951

[15] Janpan P Schmelzer B Klamrak A Tastub P Upathanpreecha T Rahman SS Production of Vespa tropica Hyaluronidase by *Pichia pastoris*. *J Fungi.* (2024) 10:854. 10.3390/jof10120854 39728350 PMC11676926

[16] Karbalaei M Rezaee SA Farsiani H. *Pichia pastoris*: a highly successful expression system for optimal synthesis of heterologous proteins. *J Cell Physiol.* (2020) 235:5867–81. 10.1002/jcp.29583 32057111 PMC7228273

[17] The Pichia System. *Pichia System History: man5 man8 | 2024 Pichia Pastoris Protein Expression Platform : Eukaryotic Protein Expression.* (2024). Available online at: https://pichia.com/science-center/james-m-cregg-ph-d/?utm_source=chatgpt.com [accessed July 28, 2025].

[18] Pokoj S Lauer I Fötisch K Himly M Mari A Enrique E Pichia pastoris is superior to E. coli for the production of recombinant allergenic non-specific lipid-transfer proteins. *Protein Express Purif.* (2010) 69:68–75. 10.1016/j.pep.2009.08.014 19733242

[19] Li P Anumanthan A Gao X-G Ilangovan K Suzara VV Düzgüneş N Expression of recombinant proteins in *Pichia pastoris*. *Appl Biochem Biotechnol.* (2007) 142:105–24. 10.1007/s12010-007-0003-x 18025573

[20] van Reis R Zydney A. Bioprocess membrane technology. *J Memb Sci.* (2007) 297:16–50. 10.1016/j.memsci.2007.02.045

[21] Chal J Pourquié O. Making muscle: skeletal myogenesis in vivo and in vitro. *Development.* (2017) 144:2104–22. 10.1242/dev.151035 28634270

[22] Pourquié O Al Tanoury Z Chal J. The long road to making muscle in vitro. *Curr Top Dev Biol.* (2018) 129:123–42. 10.1016/bs.ctdb.2018.03.003 29801528

[23] Ciofalo V Barton N Kreps J Coats I Shanahan D. Safety evaluation of a lipase enzyme preparation, expressed in *Pichia pastoris*, intended for use in the degumming of edible vegetable oil. *Regul Toxicol Pharmacol.* (2006) 45:1–8. 10.1016/j.yrtph.2006.02.001 16563586

[24] Küberl A Schneider J Thallinger GG Anderl I Wibberg D Hajek T High-quality genome sequence of Pichia pastoris CBS7435. *J Biotechnol.* (2011) 154:312–20. 10.1016/j.jbiotec.2011.04.014 21575661

[25] Hasslacher M Schall M Hayn M Bona R Rumbold K Lückl J High-level intracellular expression of hydroxynitrile lyase from the tropical rubber tree *Hevea brasiliensis* in microbial hosts. *Protein Express Purif.* (1997) 11:61–71. 10.1006/prep.1997.0765 9325140

[26] Ben-Yehezkel T Atar S Zur H Diament A Goz E Marx T Rationally designed, heterologous S. cerevisiae transcripts expose novel expression determinants. *RNA Biol.* (2015) 12:972–84. 10.1080/15476286.2015.1071762 26176266 PMC4615757

[27] Tuller T Zur H. Multiple roles of the coding sequence 5’ end in gene expression regulation. *Nucleic Acids Res.* (2015) 43:13–28. 10.1093/nar/gku1313 25505165 PMC4288200

[28] Zur H Tuller T. New universal rules of eukaryotic translation initiation fidelity. *PLoS Computat Biol.* (2013) 9:e1003136. 10.1371/journal.pcbi.1003136 23874179 PMC3708879

[29] Menuhin-Gruman I Arbel M Amitay N Sionov K Naki D Katzir I Evolutionary stability optimizer (ESO): A novel approach to identify and avoid mutational hotspots in DNA sequences while maintaining high expression levels. *ACS Synth Biol.* (2022) 11:1142–51. 10.1021/acssynbio.1c00426 34928133 PMC8938948

[30] Bergman S Tuller T. Widespread non-modular overlapping codes in the coding regions. *Phys Biol.* (2020) 17:031002. 10.1088/1478-3975/ab7083 31986496

[31] Peeri M Tuller T. High-resolution modeling of the selection on local mRNA folding strength in coding sequences across the tree of life. *Genome Biol.* (2020) 21:63. 10.1186/s13059-020-01971-y 32151272 PMC7063772

[32] Kinoshita M Kobayashi T Planells B Klisch D Spindlow D Masaki H Pluripotent stem cells related to embryonic disc exhibit common self-renewal requirements in diverse livestock species. *Development.* (2021) 148:dev199901. 10.1242/dev.199901 34874452 PMC8714072

