# Supplementary figures and images for: Yeast-derived low-purity FGF2 supports bovine ESC and MSC aggregates in suspension
Source: Front Nutr. 2025 Nov 28;12:1679490. doi: 10.3389/fnut.2025.1679490 (PMC12699982; doi:10.3389/fnut.2025.1679490)

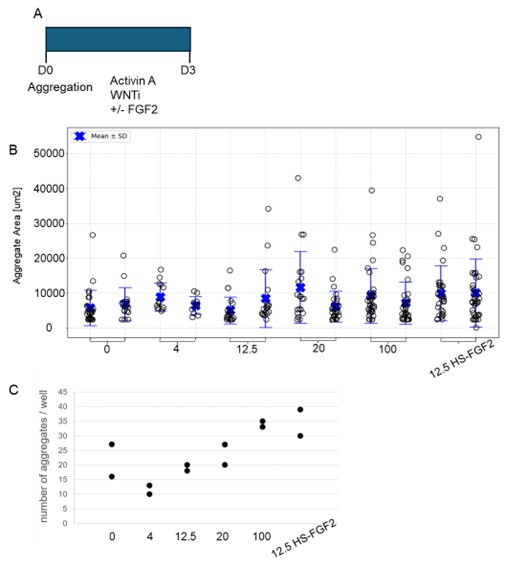

Supplement: Supplementary Figure 1 — FGF2 version and concentration effect bESC-based aggregates growth in shaking conditions. (A) Schematic representation of the experimental timeline. (B) Quantification of the number of aggregates per well on day 3, comparing different FGF2 concentrations (ng/mL) and versions. Aggregates were cultured in shaking conditions. (C) Quantification of aggregate area (μm2) on day 3 of differentiation under different FGF2 concentrations and versions. Aggregates were cultured in suspension with either heat-stable FGF2 (HS-FGF2) or standard FGF2, both commercially available at high purity. Error bars indicate standard deviation. Quantifications in both panels were performed based on bright-field images acquired from each well and analyzed using FIJI. [file Image_1.jpeg]

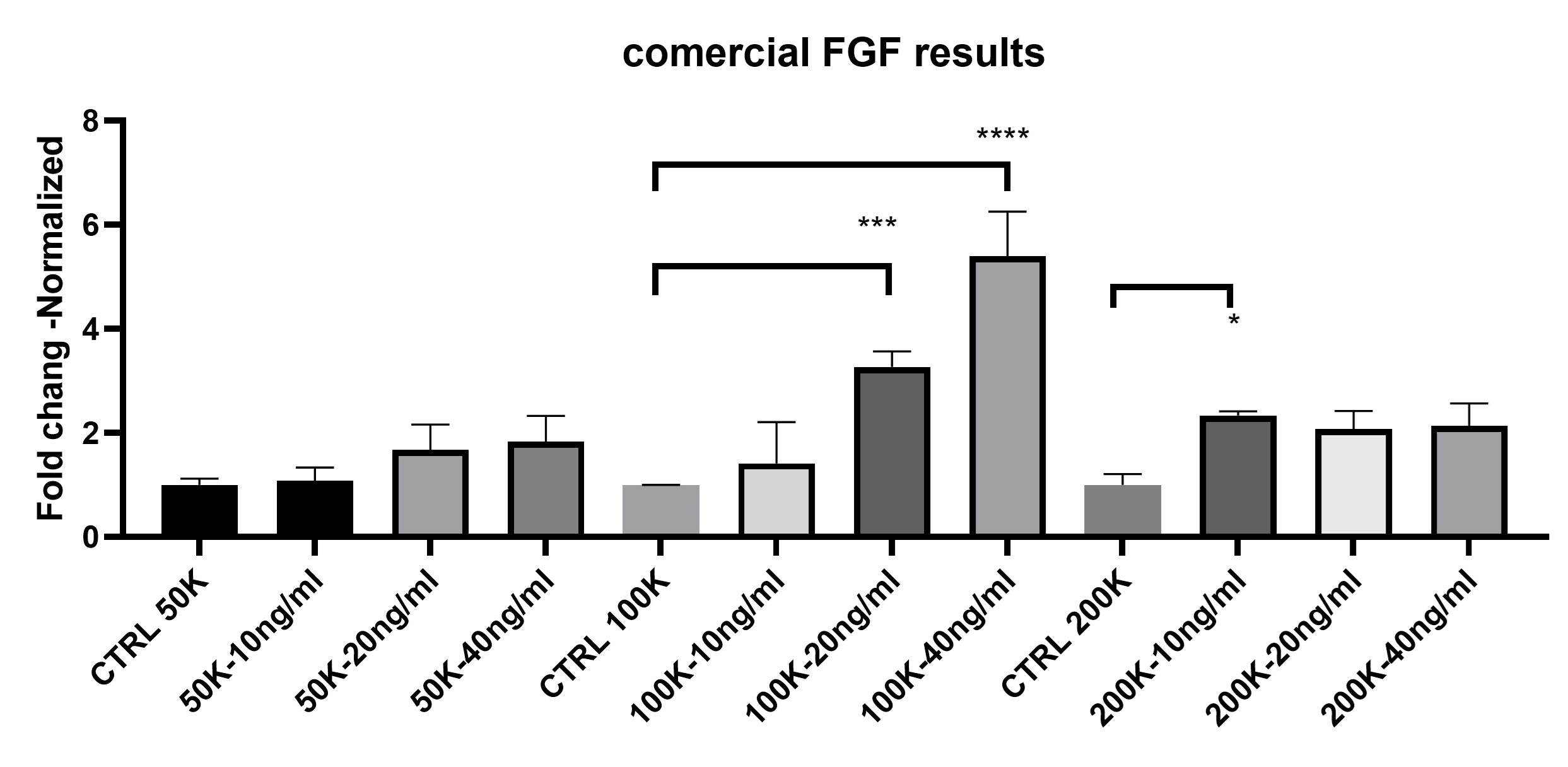

Supplement: Supplementary Figure 2 — Fold change in metabolic activity at day 7 in 3D-cultured bMSC with increasing commercial FGF concentrations. Cells were seeded in 3D at densities of 50K, 100K, or 200K and treated with 0 (control), 10, 20, or 40 ng/mL of growth factor. Alamar Blue fluorescence was measured on day 7 and normalized to values from day 2 for each seeding density. Bars represent mean ± standard deviation (SD) from 2 to 3 replicates per condition. Significant increases in normalized Alamar Blue signal were observed at 100K with 20 ng/mL (***P = 0.0003) and 40 ng/mL (****P<0.0001), and at 200K with 10 ng/mL (*P = 0.0475), compared to their respective controls (Sidak’s multiple comparisons test, α = 0.05). [file Image_2.jpeg]
